# Aortoesophageal Fistula-Induced Rare Life-Threatening Hematemesis in the Emergency Department: A Case Series and Literature Review

**DOI:** 10.7759/cureus.80980

**Published:** 2025-03-22

**Authors:** Masahiro Kobayashi, Kiyomitsu Fukaguchi, Hiroshi Koyama, Azusa Taguchi, Ichiro Sekine, Hiroshi Yamagami

**Affiliations:** 1 Department of Emergency Medicine, Shonan Kamakura General Hospital, Kanagawa, JPN; 2 Division of Critical Care, Shonan Kamakura General Hospital, Kanagawa, JPN

**Keywords:** an emergency department, aortoesophageal fistula, hematemesis, shock, syncope

## Abstract

Background and objective

Aortoesophageal fistula (AEF) is a rare but life-threatening condition. It is often challenging to promptly diagnose AEF given the diversity of chief complaints and clinical presentations at the time of initial treatment. In some cases, upper gastrointestinal endoscopy is performed, and emergency surgery is delayed, or the disease is judged to be mild and rapidly worsens, ultimately resulting in the patient's death. In this study, we aimed to investigate the clinical characteristics of AEF in patients at the time of emergency department visits, which were only partially described in previous studies.

Methods

We retrospectively reviewed the medical records of patients diagnosed with AEF from May 2013 to May 2023 at a tertiary medical center in Japan.

Results

Six patients were diagnosed with AEF during the study period. The median age of the patients was 75.5 years, and four were females. All six patients had the chief complaint of hematemesis; three (50%) had bright red hematemesis and four (67%) had a relatively small amount of hematemesis. Two (33%) experienced syncope. Three (50%) were clinically diagnosed with shock upon arrival at the hospital. Four (67%) had a history of post-aortic surgery, aortic aneurysm, or esophageal cancer.

Conclusions

AEF should be considered in patients with hematemesis, especially those with distinctive underlying diseases, shock, or syncope, even if hematemesis only involves a small amount of blood or is bright red.

## Introduction

Aortoesophageal fistula (AEF) is a rare and fatal emergent disease that causes massive hematemesis. The incidence of AEF has increased in recent years due to improved survival rates for esophageal cancer and an increase in endovascular graft aortic repair procedures [1]. However, diagnosing AEF can be challenging, and the prognosis of patients with delayed diagnosis is poor for several reasons [2]. Firstly, clinical manifestations of the condition are diverse. While the classic Chiari’s triad encompasses chest pain, sentinel hemorrhage, and massive hematemesis after an asymptomatic period, previous reports suggest that only around 20% of patients have all three [3]. Second, while patients with AEF often visit the emergency department (ED) with hematemesis [4], it is not yet known how to differentiate AEF from other hematemesis-causing illnesses [5]. Endoscopy is sometimes performed first, delaying the performance of other imaging studies.

A previous study has reported that approximately 30% of patients who initially appeared mildly ill with a small amount of hematemesis developed fatal massive hematemesis within six hours when the diagnosis was delayed [2]. While timely diagnosis in the ED is essential, there are scarce reports in the literature on the clinical presentation of AEF at the ED. In light of this, we aimed to demonstrate the clinical characteristics of patients diagnosed with AEF at the time of initial care in the ED of a tertiary medical center over 10 years. We also engage in a review of the related literature.

## Materials and methods

We retrospectively identified patients diagnosed with AEF at Shonan Kamakura General Hospital (Kamakura, Kanagawa Prefecture, Japan) between May 2013 and May 2023. Cases were identified using the hospital’s medical record system, searching for patients with the diagnosis of AEF based on code number I772 from the International Classification of Diseases, 10th Revision (ICD-10). We included patients who presented to the ED and were subsequently diagnosed with AEF. We excluded cases where AEF was incidentally detected during routine follow-ups for underlying conditions without symptoms, as well as cases previously diagnosed at another hospital and referred specifically for surgical intervention.

We reviewed medical records for chief complaints, current medical history, symptoms, characteristics and volume of hematemesis, vital signs, blood test results, chest radiographs, underlying medical conditions, treatment course, and outcomes. We defined shock as shock index >1, elevated lactate levels (≥2.0 mmol/L), and physical findings suggestive of shock, such as peripheral coldness, cold sweats, and mottled skin. A widened mediastinum was defined as a width of >80 mm on anteroposterior chest radiographs [6]. Informed consent was obtained from all patients or their surrogates.

## Results

Six of the 463,260 patients who visited the ED over 10 years were diagnosed with AEF. The clinical characteristics and course of the cases are summarized in Tables 1-2.

**Table 1:** Clinical characteristics BP: blood pressure; BUN: blood urea nitrogen; Cre: creatinine; Hb: hemoglobin; HR: heart rate; Lac: lactate; TEVAR: thoracic endovascular aortic repair; WBC: white blood cells

Sex	Age, years	Symptoms	Background and medical history	Vital signs		Physical examination	Blood test					Characteristics and amount of hematemesis	Chest X-ray mediastinal diameter, mm
				BP, mmHg	HR, beats/min		WBC, /μL	Hb, g/dL	BUN, mg/dL	Cre, mg/dL	Lac, mmol/L		
F	75	Hematemesis, dysphagia	Unknown	130/98	123	No ocular conjunctival pallor, no abdominal tenderness	12,400	11.8	24.1	0.91	1.91	One cup, bright	77
														F	85	Hematemesis	Post-TEVAR	103/51	108	Pale face, peripheral coldness, cold sweats	29,600	7.4	26.6	0.53	No records	30-cm pool of blood	99
F	87	Hematemesis, syncope, melena	Post-aortic surgery	64/40	88	Peripheral coldness, livedo reticularis, ocular conjunctival pallor	19,300	5.6	34.8	1	No records	Small amount	119														
														F	64	Hematemesis	Takayasu disease	66/31	88	No records	10,100	8.4	49.8	3.41	No records	Bright	60
M	76	Hematemesis	Esophageal cancer	118/59	82	Ocular conjunctival pallor, no abdominal tenderness	13,200	5.8	19.7	0.61	No records	One cup	73														
														M	74	Hematemesis, syncope	Unknown	88/47	95	Cold sweats, peripheral coldness	8,300	11	16.6	1.01	2.51	Bright, about 50 mL	123

**Table 2:** Clinical course CT: computed tomography; ED: emergency department

Sex	Age, years	Diagnostic procedure	Time to diagnosis from ED arrival	Course of treatment	Outcome at discharge
F	75	Upper gastrointestinal endoscopy was performed first, diagnosed with contrast-enhanced CT afterward	7 hours	Discharged alive after aortic stent graft placement, and additional stenting	Alive
F	85	Diagnosis by upper gastrointestinal endoscopy	5 hours	In poor general condition and died inoperable	Died
F	87	A contrast-enhanced CT was performed first, and then, combined with endoscopic findings, a diagnosis was made	4 hours	Went into cardiopulmonary arrest before the intervention	Died
F	64	Hematemesis during upper gastrointestinal endoscopy, subsequent diagnosis by contrast-enhanced CT	2.5 hours	Hemorrhaged blood and died a few weeks after aortic stent graft placement	Died
M	76	Upper gastrointestinal endoscopy was performed first, diagnosed with contrast-enhanced CT afterward	15 hours	Died a few weeks after aortic stent graft placement	Died
M	74	Surgical intervention without endoscopy after contrast-enhanced CT and diagnosis of primary aortic aneurysm	1 hour	Underwent aortic stent graft placement and total esophagectomy the next day and was discharged alive	Alive

**Figure 1:**
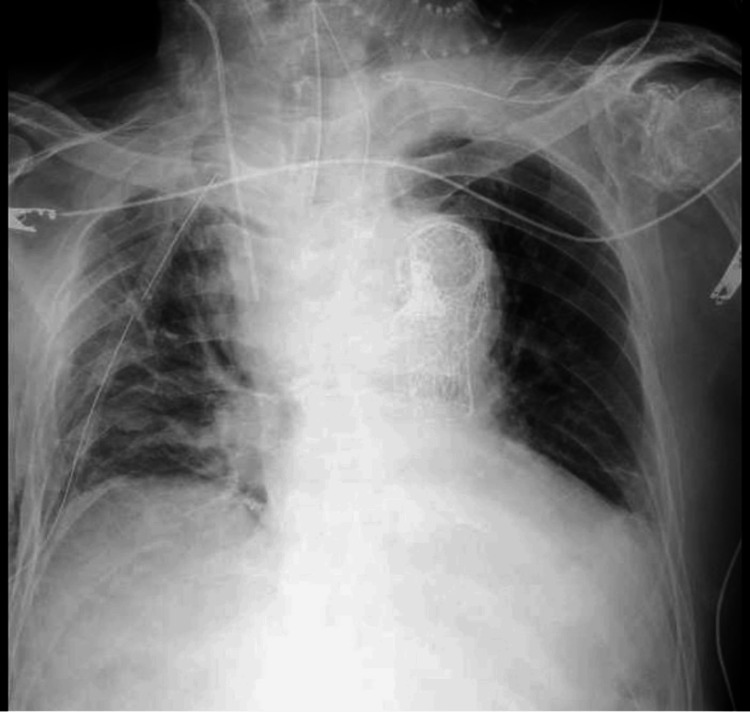
Portable chest X-ray Portable chest X-ray showing significant mediastinal widening in a patient with aortoesophageal fistula (AEF), suggesting possible aortic pathology and associated complications

The median age of the patients was 75.5 years, and four were female. The chief complaint in all six was hematemesis, and none had chest pain. In addition, two patients had hematemesis with syncope. Hematemesis was described as bright red in three patients, and the amount of hematemesis was relatively small in four patients with shock, who described it as "a cup," approximately 50 mL, a small amount, or a "cup of blood." Four patients experienced shock upon arrival at the hospital. Two patients had ocular conjunctival pallor. Blood tests revealed an elevated blood urea nitrogen/creatinine ratio in two patients, elevated white blood cells in four patients, and anemia in four patients; however, none of the findings were specific to AEF. Three patients had a widened mediastinum on chest X-ray, as shown in Figure 1.

**Figure 2:**
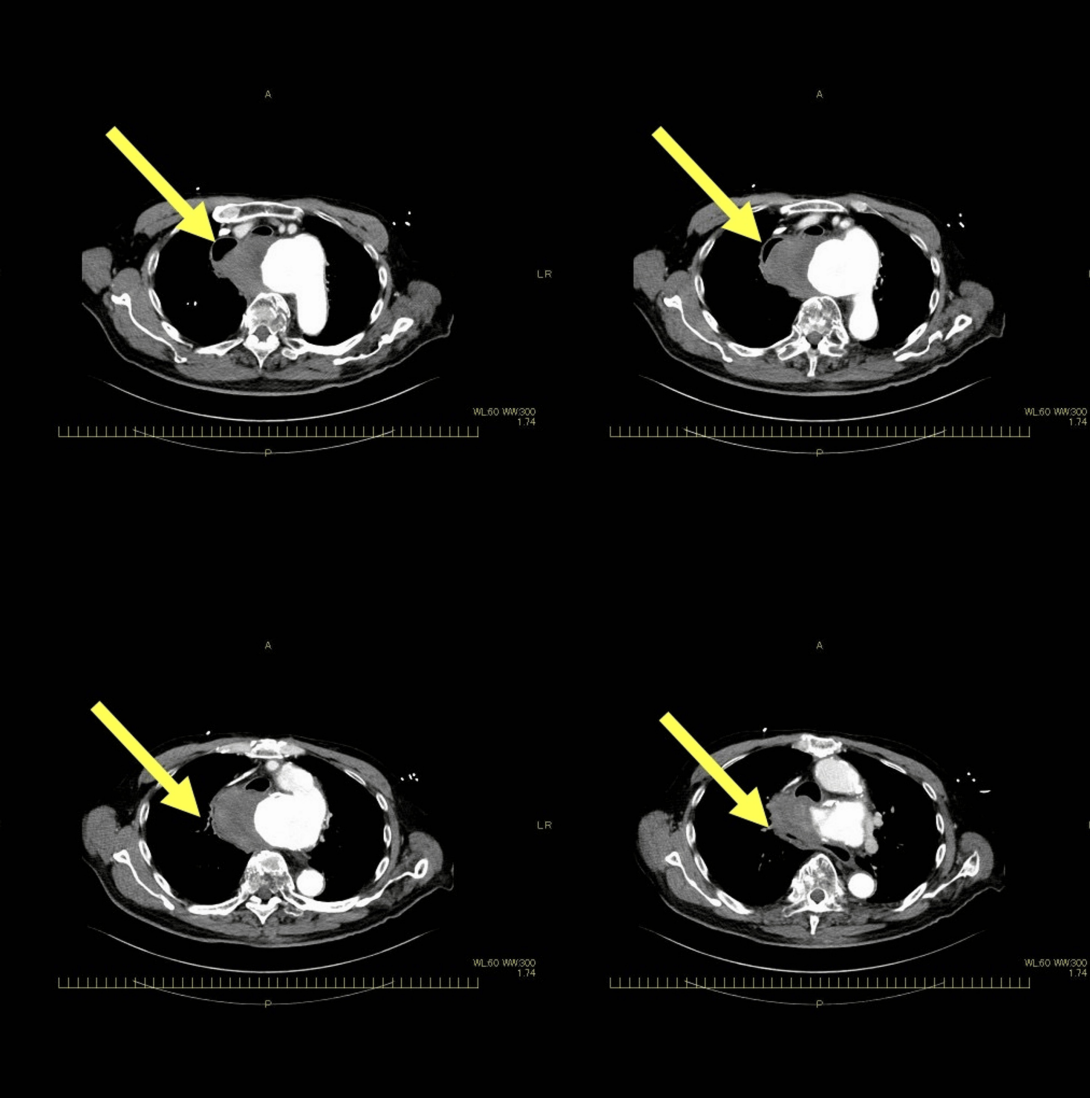
Contrast-enhanced CT Axial contrast-enhanced CT image showing a hematoma from an aortic aneurysm compressing the esophagus, suggestive of aortoesophageal fistula CT: computed tomography

**Figure 3:**
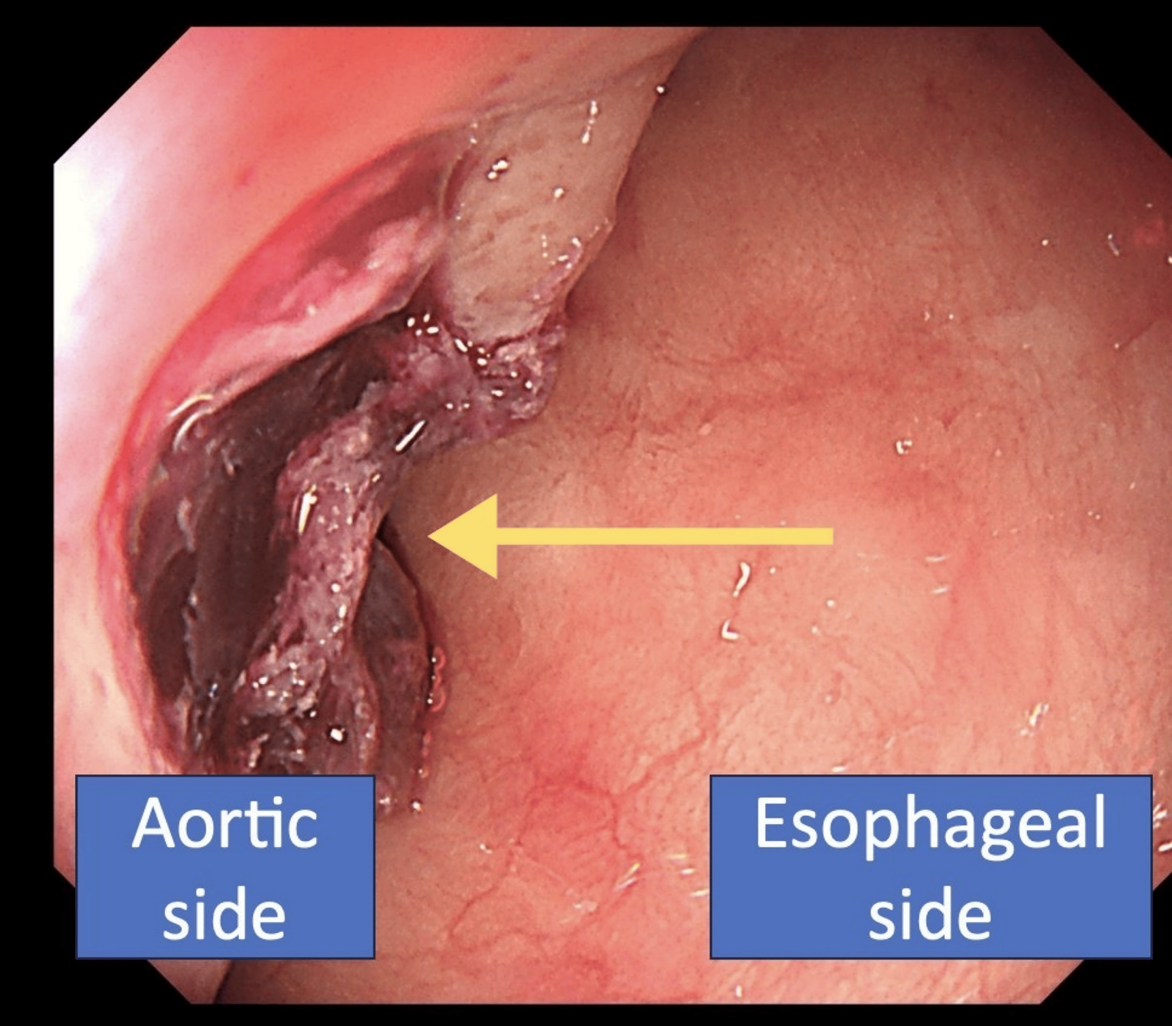
Upper gastrointestinal endoscopy Upper gastrointestinal endoscopy showing a laceration with a suspected fistula and blood clot, indicative of an aortoesophageal fistula. The right side corresponds to the esophageal lumen, while the left side is adjacent to the aorta

Concerning medical history and background, two patients had a history of previous aortic surgery, one had previous esophageal cancer, and one had previous Takayasu disease, while two patients had no preexisting medical conditions. The median time from ED arrival to diagnosis was 4.5 hours. One patient underwent surgical treatment based on contrast-enhanced CT findings alone, without upper gastrointestinal endoscopy, as shown in Figure 2. As shown in Figure 3, upper endoscopy was performed first in four patients to identify the cause of upper gastrointestinal bleeding. Four underwent emergency aortic surgery after the AEF diagnosis, and two were discharged. To summarize, in this study, 67% of the patients with AEF had distinctive underlying diseases at the time of their ED visit, and half of the patients had bright red hematemesis or hemodynamic instability despite only a small amount of hematemesis.

## Discussion

In this case series, it was considered important to differentiate AEF from other hematemesis-causing diseases by focusing on bright hematemesis, shock, or syncope despite the small amount of bleeding and background disease. To validate as many cases of AEF as possible, we reviewed published literature to examine AEF symptoms and underlying diseases. We used the term “aortoesophageal fistula” to conduct a search in the MEDLINE database for articles published up to January 2024.

We identified 50 well-informed references containing information on patients diagnosed with AEF and reviewed their main symptoms and underlying diseases. Table 3 shows the results of the literature analysis. Chiari's triad was defined as chest pain, sentinel hemorrhage, and repeated hematemesis during the asymptomatic period. Shock was defined as hypotension, shock index >1, and hemodynamic instability with peripheral coldness, cold sweats, and mottled skin.

**Table 3:** Main symptoms and underlying diseases based on 50 references AEF: aortoesophageal fistula; TEVAR: thoracic endovascular aortic repair

Characteristics of AEF	% (n/total cases)
Main symptoms	
Hematemesis	83% (226/272)
Shock	45.5% (122/268)
Chest pain, abdominal pain, back pain	37.9% (101/266)
Fever	25.1% (67/266)
Chiari’s triad	18.1% (48/265)
Melena	11.7% (7/60)
Syncope	8.3% (5/60)
Underlying diseases or treatment histories	
Foreign body ingestion	23.3% (14/60)
TEVAR	21.7% (13/60)
Aortic aneurism	18.3% (11/60)
Esophageal cancer	10% (6/60)

Chief complaint and symptoms

Chest pain, sentinel hemorrhage, and massive hematemesis after an asymptomatic period are classically referred to as the Chiari triad. However, in previous reviews, Chiari's triad occurred in only 11% to 20% of cases [2,3]. In this study, all patients also had hematemesis as their chief complaint, but none of them complained of chest pain, suggesting that few patients had the triad. In addition, half of them had fresh blood hematemesis, and four of the six patients presented with shock, syncope, or both. Table 3 shows the same trend. In the review we conducted, only 18.1% presented Chiari’s triad, but 45.5% presented shocks.

Hollander et al. stated that AEF can be diagnosed bedside in cases of “bright” bleeding [7], but there have been no additional studies to support this statement, and it is not well-discussed in recent literature. In our case series, the nature of hematemesis was recorded in three of six cases; all three cases had bright red hematemesis. In the two cases in which bleeding volumes were described, the patients went into shock with a relatively small amount of blood loss. Hematemesis with shock is commonly caused by esophageal varices or bleeding from pathological gastric or duodenal abnormalities. Esophageal varices typically produce dark red hematemesis because the bleeding originates from a vein. Bleeding from the stomach or duodenum can be arterial in origin but is more likely to be dark in color because the blood is exposed to stomach acid [7]. Thus, bright arterial hematemesis suggests that bleeding from the gastrointestinal tract originates from a major arterial branch proximal to the stomach and that relatively small amounts of hematemesis due to sentinel bleeding may be associated with shock and syncope.

Etiology and medical history

Of the six hematemesis cases in our case series, two occurred after aortic surgery, one was due to a primary aortic aneurysm, two because of esophageal cancer, and one was due to aortic disease arteritis (Takayasu disease). Although the prevalence of AEF is not yet clearly known, one study suggested an annual incidence of 0.007 per million [8]. There are two types, primary and secondary, with the latter reported to be 10 times more common [2]. The latter occurs after invasive treatments, such as thoracic aortic or esophageal surgery or stenting. Among primary AEFs, the most common cause is reportedly atherosclerotic aortic aneurysm, accounting for approximately 50% [9], while foreign body ingestion and esophageal cancer represent approximately 20% each [10].

In a review of AEF over the past 10 years, 61 of 172 cases occurred after aortic surgery; 45 were due to primary aortic aneurysms; 25 resulted from foreign body ingestion; and 23 were due to thoracic cancers [11]. In our review of the literature (Table 3), foreign body ingestion was the most common type of ingestion (23.3%), followed by post-TEVAR (21.7%). This may be due to the possibility of more foreign body ingestion in younger patients when it comes to case reports. Considering these factors, medical and treatment histories such as aortic surgery, primary aortic aneurysms, foreign body ingestion, and thoracic cancers may provide a possible clue for suspecting AEF in patients with hematemesis.

Diagnosis and course of treatment

Gastrointestinal endoscopy is usually recommended to diagnose gastrointestinal bleeding. However, endoscopy for AEF can remove the occlusive periaortic hematoma and cause fatal bleeding [12]. The sensitivity of endoscopy is 25-80%, and the sensitivity of contrast CT is up to 93% [13]. Considering these facts, we believe that contrast-enhanced CT should be performed promptly before endoscopy when AEF is considered. In this study, only one case was diagnosed using contrast-enhanced CT alone, and emergency upper gastrointestinal endoscopy was performed first in four cases. Distinguishing AEF from upper gastrointestinal bleeding can be difficult when a patient presents with hematemesis as the chief complaint. The fatality rate of AEF without treatment was reportedly as high as 100 %; even with surgical intervention, it was reportedly as high as 75% [14]. In this study, operations were performed in four cases, but only two patients survived, which is consistent with previous studies showing poor prognosis even after surgical procedures [15]. It is a difficult condition to save lives with, even if surgery can be done, but if it cannot be diagnosed, there is no chance. So, it's important to get it into the differential first and diagnose it correctly.

This study has several limitations. Firstly, it was conducted at a single institution and the sample was small (only six cases). However, it is an important study that focuses on the characteristics of rare and potentially fatal AEF experienced in the ED. Second, it is possible that some of the critical patients who died before or shortly after arrival at the hospital were not diagnosed with AEF. To recognize and save such cases, it will be necessary to accumulate reports of more such cases and enhance the recognition of AEF in the future.

## Conclusions

When examining a hematemesis patient in the ED, AEF should be considered in the setting of a history of aortic aneurysm, aortic surgery, or esophageal cancer, as well as in the presence of hemodynamic instability despite a small amount of bleeding, and in the presence of bright red hematemesis. We recommend that contrast-enhanced CT precede endoscopy during the initial treatment.
